# Tracing the Geographical Origins of Dendrobe (*Dendrobium* spp.) by Near-Infrared Spectroscopy Sensor Combined with Porphyrin and Chemometrics

**DOI:** 10.1155/2020/8879957

**Published:** 2020-09-12

**Authors:** Chaogeng Lv, Yali He, Chuanzhi Kang, Li Zhou, Tielin Wang, Jian Yang, Lanping Guo

**Affiliations:** State Key Laboratory Breeding Base of Dao-di Herbs, National Resource Center for Chinese Materia Medica, China Academy of Chinese Medical Sciences, Beijing 100700, China

## Abstract

Dendrobe (*Dendrobium* spp.) is a traditional medicinal and edible food, which is rich in nutrients and contains biologically active metabolites. The quality and price of dendrobe are related to its geographical origins, and high quality dendrobe is often imitated by low quality dendrobe in the market. In this work, near-infrared (NIR) spectroscopy sensor combined with porphyrin and chemometrics was used to distinguish 360 dendrobe samples from twelve different geographical origins. Partial least squares discriminant analysis (PLSDA) was used to study the sensing performance of traditional NIR and tera-(4-methoxyphenyl)-porphyrin (TMPP)-NIR on the identification of dendrobe origin. In the PLSDA model, the recognition rate of the training and prediction set of the TMPP-NIR could reach 100%, which was higher than the 91.85% and 91.34% of traditional NIR. And the accuracy, sensitivity, and specificity of the TMPP-NIR sensor are all 1.00. The mechanism of TMPP improving the specificity of NIR spectroscopy should be related to the *π*-*π* conjugated system and the methoxy groups of TMPP interact with the chemical components of dendrobe. This study reflected that NIR spectrum with TMPP sensor was an effective approach for identifying the geographic origin of dendrobe.

## 1. Introduction

Dendrobe is the general name of the medicinal plants from the *Orchidaceae* family, *Dendrobium* genus, which mainly includes *D. nobile* Lindl., *D. candidum* Wall., *D. fimbriatum* Hook, and *D. chrysotoxum* Lindl. [1], and is regarded as high-end medicinal and edible medicinal herb in China [2, 3]. Dendrobe was first recorded in “Shen Nong's Herbal Classic” [4], and it was also recorded in “Famous Doctors,” “Compendium of Materia Medica,” “Chinese Medicine History,” and “People's Republic of China Pharmacopoeia,” for more than 2000 years [1, 5]. These ancient documents record about 76 kinds of dendrobe and more than 50 kinds can be processed into a valuable edible medicinal material, such as tea, beverage, supplement food, and Chinese herbal medicine [6]. Modern studies have found that it contains many active ingredients like polysaccharides, flavonoids, and alkaloids, which have antioxidation, antitumor, immune regulation, antifatigue, treatment of diabetes, and other pharmacological effects [7, 8]. The dendrobe from Huoshan, Anhui province of China, especially, has the best quality and its price is also much higher than that of dendrobe from Zhejiang, Yunnan, Guangxi, Guangdong, and other places of China [9, 10]. At the same time, due to the large market demand and the confusion of varieties and producing area of dendrobe, businessmen often use low quality dendrobe as high quality dendrobe to seek profits, such as dendrobe from Yunnan and Zhejiang posing as dendrobe from Anhui. Therefore, it is very important to identify the species and habitats of dendrobe quickly and accurately.

Up to now, various methods have been reported to distinguish the species and habitats of dendrobe. It is the most direct method to identify dendrobe according to its shape, color, texture, smell, and other characteristics by seeing, touching, smelling, and tasting [11, 12]. This method is simple, fast, and intuitive, but it relies too much on personal experience. Microscopic identification can also be used to distinguish dendrobe species with different microscopic characteristics but similar appearance [12]. The disadvantage of this method is that it is more complicated to process samples, and it can only identify varieties with obvious differences in microstructure. In order to accurately distinguish the dendrobe, DNA molecular labeling techniques are used to identify dendrobe with unobvious microscopic features [13, 14]. In recent years, with the development of chromatography technology, it has also been applied to the identification of dendrobe [15, 16], like high-performance liquid chromatography (HPLC) and HPLC-mass spectrum (HPLC-MS), which can provide a large amount of chemical composition information for the identification of dendrobe origin [17–21]. DNA molecular labeling and chromatography have the advantages of reproducibility, high separation efficiency, and high sensitivity, but they have high technical requirements, expensive analysis costs, and long analysis time. In addition, ultraviolet spectroscopy and infrared spectroscopy are commonly used to identify dendrobe from different origins, in order to reduce the detection cost and increase the detection speed [22, 23], but the specificity of these spectroscopic methods is poor. Among them, near-infrared (NIR) spectroscopy is a commonly used analytical method to study the qualitative or quantitative information of the internal molecular structure of substances. It is a mature method for food and drug analysis due to its advantages of less dosage, being fast, being nondestructive, and high sensitivity [24–26]. And it had been used to distinguish *D. nobile* Lindl. from Yunnan and Zhejiang provinces [27], and different species of dendrobe from 7 producing areas [28]. However, the accuracy of NIR is still not enough, so it is necessary to increases the accuracy of identification for NIR.

To overcome the subjective error of traditional methods and solve the problems of long time, expensive cost, and high technical requirements of DNA molecular labeling and chromatography, the sensing method of near-infrared spectroscopy combined with porphyrin and chemometrics was proposed in this work. The combined use of spectroscopy and chemometric analysis has shown great success in some studies, such as the identification of authenticity, variety, and geographic origin [29–31]. The combination of near-infrared spectroscopy and chemometrics can carefully examine and interpret all the complex information generated by a single spectrum, such as cluster analysis (CA), principal component analysis (PCA), and partial least squares discriminant analysis (PLSDA) [32–35]. In addition, the combination of near-infrared and chemometrics information diversity solves the problem of quality information extraction and interference signal removal, which provides favorable support for accurate modeling and identification of dendrobe.

In this study, the potential of traditional NIR and porphyrin-NIR sensor for the geographical origins tracing of dendrobe was tested and discussed by chemometrics. The results indicated that porphyrin can increase the specificity of NIR spectrum in the habitats identification of dendrobe, which mean that this work provides a new idea for the geographical origins tracing by NIR.

## 2. Materials and Methods

### 2.1. Materials and Apparatus

A total of 360 batches of fresh dendrobe were collected from 12 different geographical origins in China, including three varieties of dendrobe. These varieties were *D. huoshanense*, *D. nobile* Lindl., and *D. officinale*. Tera-(4-methoxyphenyl)-porphyrin (TMPP) was purchased from Sigma-Aldrich Trading Co., Ltd. (Shanghai, China). Methanol (analytical reagent) was bought from Sinopharm Chemical Reagent Co., Ltd. (Shanghai, China). NIR spectrum of traditional dendrobe and TMPP-dendrobe mixture were collected using an Antaris II Fourier Transform Near-Infrared Spectrometer (Thermo Nicolet Company, USA) equipped with an InGaAs detector, using FW-100 high-speed universal pulverizer (Tianjin Test Instrument Co., Ltd., Tianjin, China) to crush dendrobe medicinal materials and using DZ-1BC|| vacuum drying oven (Tianjin Test Instrument Co., Ltd., Tianjin, China) to vacuum dry the samples.

### 2.2. Collection and Processing of Dendrobe Samples

**Figure 1:**
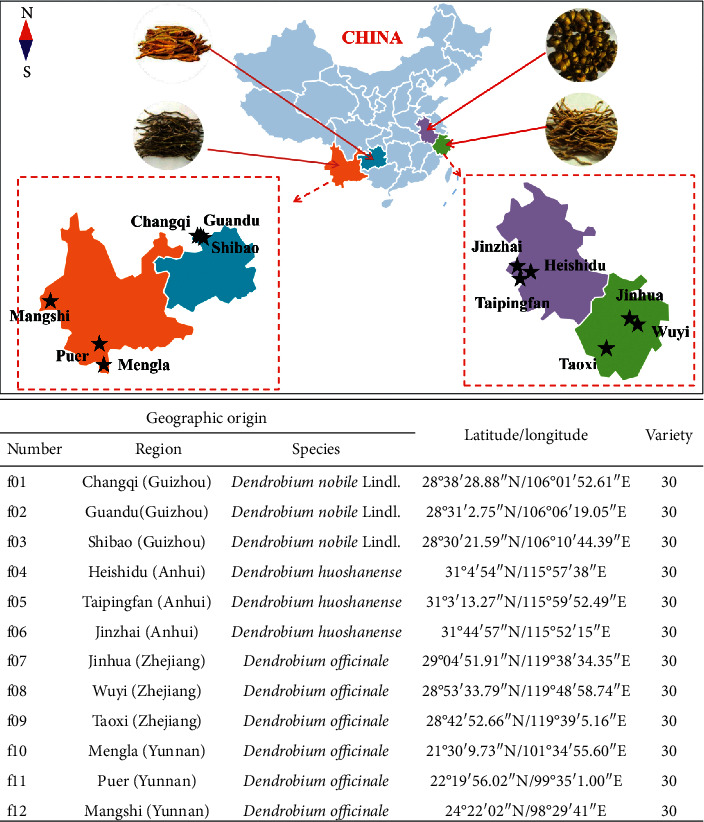
Information about dendrobe from different origins.

In order to determine the geographic origin of dendrobe, we invited professional researchers to collect it from local dendrobe growers and all samples collected from October to December 2019. The specific information of dendrobe collected is shown in Figure 1. Fresh dendrobe was cleaned with ultrapure water and cut into small sections, then dried at 60°C until the moisture meets the requirements and, finally, sealed and stored for subsequent experiments.

### 2.3. Spectra Acquisition

The dried dendrobe samples were finely ground into powder and screened by a stainless steel standard inspection sieve with a size of 75 *μ*m aperture, then dried under vacuum at 60°C for 24 hours, and then stored in a desiccator. The dendrobe sample and TMPP powder sample were thoroughly mixed at a mass ratio of 3 : 1, and a methanol solution with a material-to-liquid ratio of 10 : 1 was added, ultrasonicated for 30 min, and filtered; then mixture was freeze-dried. The dried mixed sample was subjected to the same method as above to dry and store. The NIR spectra of dendrobe and TMPP-dendrobe mixtures were collected using Antaris II Fourier Transform Near-Infrared Spectrometer equipped with InGaAs detector. The collection conditions were 10 scans and the spectrum range was 4000∼10000 cm^−1^; resolution was 8 cm^−1^; relative humidity was (45 ± 1) %; and temperature was (25 ± 1)°C. Dendrobe from each producing area was measured and a total of 720 samples spectra were collected.

### 2.4. Data Processing by Chemometrics

The collected spectral data uses MATLAB 7.10.0 (R2010a) (Mathworks, Natick, MA, USA) software and chemometric algorithm (Partial least squares discriminant analysis, PLS-DA) to process. PLS-DA is a multivariate statistical analysis method for discriminant analysis; latent variables (LVs), accuracy, sensitivity, and specificity are used to evaluate the reliability of the model. LVs are selected through 10-fold cross-validation to optimize this model to obtain the best results. The accuracy, precision, and sensitivity of the model are calculated as follows:

(1) $$\begin{array}{c} \text{Accuracy}=\frac{\left(\text{TP}+\text{TN}\right)}{\left(\text{TP}+\text{TN}+\text{FP}+\text{FN}\right)},\\ \text{Sensitivity}=\frac{\text{TP}}{\left(\text{TP}+\text{FN}\right)},\\ \text{Specificity}=\frac{\text{TN}}{\left(\text{TN}+\text{FP}\right)}, \end{array}$$

where TP represents true positive; TN represents true negative; FN represents false negative; and FP represents false positive, respectively.

## 3. Results and Discussion

### 3.1. Near-Infrared Spectrum Sensor of Dendrobe

**Figure 2:**
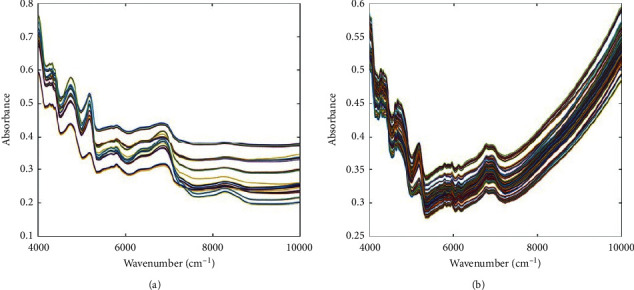
The original NIR spectra of dendrobe (a) and dendrobe with TMPP (b).

The average absorption spectra of the dendrobe with or without TMPP are shown in Figure 2. The peak absorption value in the range of 4000–10000 cm^−1^ was passed through NIR to obtain the composite spectrum of the absorption peak of different types of compounds in dendrobe, such as flavonoids, polysaccharides, dendrobine, and amino acids [36, 37]. Generally, the chemical composition of dendrobe directly affects its spectral characteristics. The differences in the chemical composition of dendrobe from different origins and varieties will also be shown by the differences in chemical composition so that the quality can be judged by the differences in spectral characteristics. It can be seen from Figure 2 that the traditional spectrogram overlaps into 5 parts, and the spectra overlap severely, which is difficult to visualize, while the spectrogram with TMPP is more scattered and facilitates visualization. The result revealed that TMPP can increase the specificity of NIR spectra through changing the shape and intensity of the spectrum, which may be related to the interaction between dendrobe and porphyrin.

### 3.2. PLSDA Analysis of Dendrobe Samples from Different Habitats

**Figure 3:**
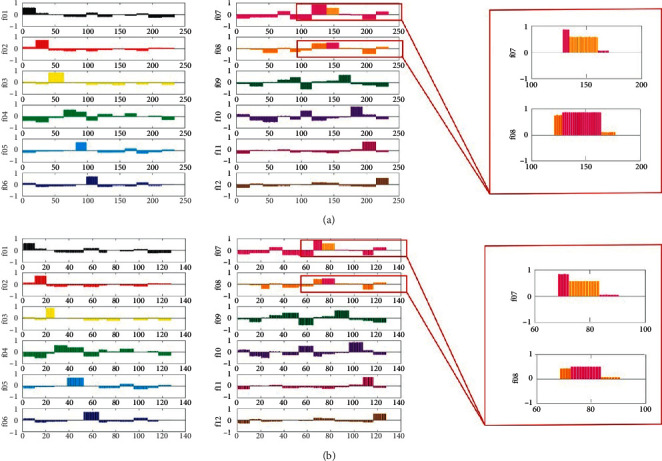
Dummy codes plots of the PLSDA model for traditional dendrobe NIR spectra. (a) Training sample. (b) Prediction sample.

**Figure 4:**
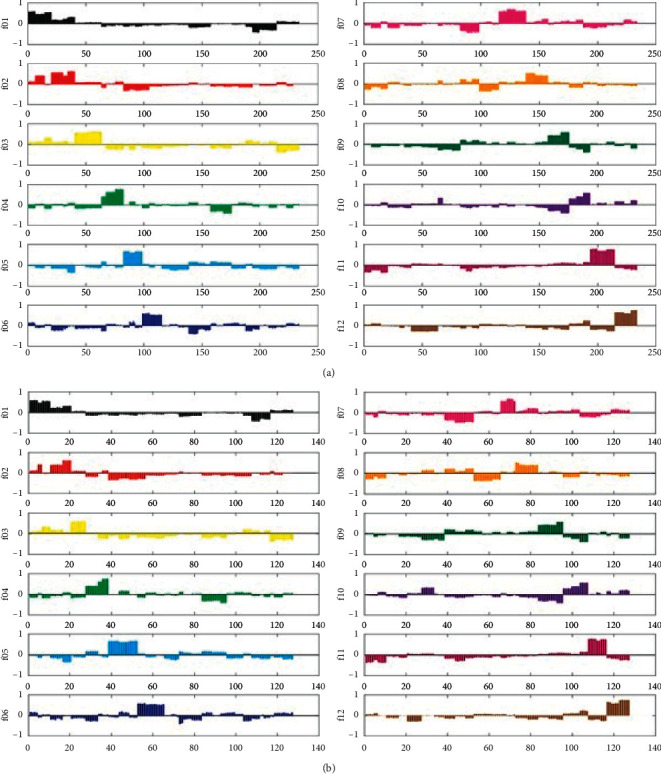
Dummy codes plots of the PLSDA model for dendrobe NIR spectra with TMPP. (a) Training sample. (b) Prediction sample.

In order to classify the dendrobe from different geographical origins, a discriminant model was established by supervised partial least squares analysis, and the discriminant analysis of dendrobe from 12 geographical origins was carried out. The class feature matrix in PLSDA is represented by the class of different classes of virtual vectors with encoded virtual vectors. The training and prediction set of PLSDA were divided randomly as shown in Table 1. The results (Figure 3) of NIR spectrum without TMPP displayed that 19 samples from 139^th^ to 157^th^ samples of the f08 training sample were misjudged to the f07 sample, and 11 samples from the 73^rd^ to the 83^rd^ predicted samples were also wrongly assigned to group f07 from f08. The accuracy of traditional NIR spectroscopy on the geographical origins discrimination could only achieve 91.85% and 91.34% in the training and prediction set, respectively (Table 2). This method caused the misclassification of dendrobe sample f08 from Wuyi County, Zhejiang Province, and dendrobe sample f07 from Jinhua City, Zhejiang Province, which might be due to the homology and their similar growth environment. However, the NIR spectra with TMPP can successfully identify 12 kinds of dendrobe from different geographical origins, the recognition rate of which can all reach 100% (Figure 4 and Table 2). Moreover, by comparing the overall accuracy, sensitivity, and specificity of traditional NIR spectra and NIR spectra with TMPP, the traditional spectra were 0.96, 0.94, and 0.90, respectively; and the corresponding values of spectra with TMPP were 1.00, 1.00, and 1.00, respectively (Table 2).

The improved discrimination performance of NIR spectroscopy sensor by TMPP could be attributed to the excellent photosensitivity of TMPP and its specific interaction with the compounds of dendrobe. Porphyrin and its derivatives have a wide range of optical absorption characteristics and good photosensitivity. In its structure, the *α*-carbon atoms of the four pyrrole subunits are interconnected by a methine bridge (=CH-) to form a *π*-*π* conjugated system, when the structure of porphyrin and its derivatives changes, its optical properties change. The chemical composition of dendrobe is complex, containing polysaccharides, flavonoids, alkaloids, amino acids, and other compounds. After porphyrin is mixed with dendrobe, the complex chemical composition can interact with porphyrin, which greatly affects the optical properties of porphyrin. And much characteristic information is obtained during NIR spectrum scanning, which improves the accuracy of this model. And the PLSDA results (Table 2) revealed that the developed NIR-based TMPP sensor was accurate and reliable. So, it was recommended that the NIR-based TMPP sensor is the first choice for the geographical origins tracing of dendrobe to obtain better accuracy.

## 4. Conclusions

In this study, 360 samples of dendrobe from 12 different geographical origins were accurately traced using NIR spectroscopy sensor combined with porphyrin and chemometric. Compared with traditional NIR spectra, the NIR spectra with TMPP can obtain more characteristic information, which greatly improves the accuracy of model identification. After adding TMPP, the accuracy rate of NIR spectra reached 100%. The possible mechanism was that the *π*-*π* conjugated system and the methoxy groups of TMPP interact with the chemical components of dendrobe, which increase the specificity of NIR spectra. This study displayed that the combination of NIR spectroscopy and TMPP is an effective method to identify the geographic origin of dendrobe. And we will conduct an in-depth study of the reaction mechanism between TMPP and the compounds of dendrobe.

## Figures and Tables

**Table 1:** Random division of training and prediction set for PLSDA analysis.

Group code	Training set		Prediction set	
	No.	Samples	No.	Samples
f01	20	1^st^-20^th^	10	1^st^-10^th^
f02	20	21^st^-40^th^	10	11^th^-20^th^
f03	23	41^st^-63^rd^	7	21^st^-27^th^
f04	19	64^th^-82^nd^	11	28^th^-38^th^
f05	16	65^th^-98^th^	14	39^th^-52^nd^
f06	17	99^th^-115^th^	13	53^rd^-65^th^
f07	23	116^th^-138^th^	7	66^th^-72^nd^
f08	19	139^th^-157^th^	11	73^rd^-83^rd^
f09	18	158^th^-175^th^	12	84^th^-95^th^
f10	18	176^th^-193^rd^	12	96^th^-107^th^
f11	21	194^th^-214^th^	9	108^th^-116^th^
f12	19	215^th^-233^rd^	11	117^th^-127^th^

**Table 2:** Geographical origins traceability performance of traditional NIR spectra and NIR spectra with TMPP.

	Traditional NIR spectra	NIR spectra with TMPP
LVs	3	3
Mistake number of training sets	19	0
Mistake number of predicting sets	11	0
Accuracy rate of training set	91.85%	100%
Accuracy rate of predicting set	91.34%	100%
Accuracy	0.96	1
Sensitivity	0.94	1
Specificity	0.90	1

## Data Availability

The data used to support this study are available from the corresponding author upon request.
